# Soluble Epoxide Hydrolase Inhibition Protected against Diabetic Cardiomyopathy through Inducing Autophagy and Reducing Apoptosis Relying on Nrf2 Upregulation and Transcription Activation

**DOI:** 10.1155/2022/3773415

**Published:** 2022-03-25

**Authors:** Qin Fang, Xiaohui Liu, Jie Ding, Zhihao Zhang, Guangzhi Chen, Tingyi Du, Yan Wang, Renfan Xu

**Affiliations:** ^1^Division of Cardiology, Department of Internal Medicine, Tongji Hospital, Tongji Medical College, Huazhong University of Science and Technology, Wuhan 430030, China; ^2^Hubei Key Laboratory of Genetics and Molecular Mechanisms of Cardiological Disorders, Wuhan 430030, China; ^3^Department of Medical Ultrasound, Tongji Hospital, Tongji Medical College, Huazhong University of Science and Technology, Wuhan 430030, China

## Abstract

**Background:**

Many patients with diabetes die from diabetic cardiomyopathy (DCM); however, effective strategies for the prevention or treatment of DCM have not yet been clarified.

**Methods:**

Leptin receptor-deficient (db/db) mice were treated with either the soluble epoxide hydrolase (sEH) inhibitor AUDA or vehicle alone. A virus carrying Nrf2 shRNA was used to manipulate Nrf2 expression in db/db mice. Cardiac structures and functions were analyzed using echocardiography and hemodynamic examinations. Primary cardiomyocytes cultured under high glucose and high fat (HGHF) conditions were used to conduct *in vitro* loss-of-function assays after culture in the presence or absence of AUDA (1 *μ*M). Fluorescence microscopy-based detection of mCherry-GFP-LC3 was performed to assess autophagic flux.

**Results:**

The sEH inhibitor AUDA significantly attenuated ventricular remodeling and ameliorated cardiac dysfunction in db/db mice. Interestingly, AUDA upregulated Nrf2 expression and promoted its nuclear translocation in db/db mice and the HGHF-treated cardiomyocytes. Additionally, AUDA increased autophagy and decreased apoptosis in db/db mice heart. Furthermore, the administration of AUDA promoted autophagic flux and elevated LC3-II protein level in the presence of bafilomycin A1. However, AUDA-induced autophagy was abolished, and the antiapoptotic effect was partially inhibited upon Nrf2 knockdown.

**Conclusion:**

Our findings suggest that the sEH inhibitor AUDA attenuates cardiac remodeling and dysfunction in DCM via increasing autophagy and reducing apoptosis, which is relevant to activate Nrf2 signaling pathway.

## 1. Background

Diabetic cardiomyopathy (DCM) is characterized by cardiac dysfunction in the absence of valvular disease, coronary artery disease, and other cardiovascular risk factors, such as dyslipidemia and hypertension [1]. DCM is a major cause of increased morbidity and mortality in diabetic patients worldwide [2]. Despite a massive increase in numbers of preclinical and clinical studies on DCM over the past decade, the pathogenesis of this condition remains unclear. Among new antidiabetic agents, dipeptidyl peptidase 4 inhibitors do not lower the risks of cardiovascular disease and death in type 2 diabetic patients compared to the risk in controls [3]. On the contrary, sodium-glucose cotransporter 2 (SGLT2) inhibitors were shown to decrease the risk of cardiovascular disease in type 2 diabetic patients compared to placebo [4–6]. However, a clinical trial showed that treatment with the SGLT2 inhibitor dapagliflozin reduced the risk of cardiovascular disease-related death in patients with or without type 2 diabetes [7], suggesting that the drug reduces the risk of cardiac complications independently of its blood glucose-lowering properties. Therefore, further understanding of the pathological mechanisms of DCM in type 2 diabetic patients is required, as effective preventive and therapeutic strategies for DCM remain elusive [8].

Type 2 diabetes is featured with hyperglycemia, insulin resistance, and obesity, which eventually lead to cardiomyocyte apoptosis and cardiac dysfunction [9]. Emerging studies suggest that impaired myocardial insulin signaling [10], calcium homeostasis [10], mitochondrial dysfunction [11], reduced nitric oxide bioavailability [12], increased oxidative stress [13], impaired microvascular dysfunction [14], and a myriad of other metabolic cardiac abnormalities [15] are involved in DCM progression. However, the pathophysiological mechanisms that affect type 2 diabetic patient heart were poorly understood. Basal constitutive autophagy was shown to play a cytoprotective role in the heart by maintaining cell function under stress conditions [16]. Autophagy is a highly conserved mechanism of intracellular protein and organelle recycling that regulates cell survival and function [17]. It plays an essential role in maintaining organelle function and protein quality by removing damaged organelles and protein aggregates [18]. Recent evidence suggests that inhibited cardiomyocyte autophagy, elevated apoptosis, and oxidative stress are observed in DCM [19, 20]. Moreover, increased autophagy has been shown to protect cardiomyocytes against the hyperglycemia-induced oxidative stress and apoptosis [21, 22].

In addition to autophagy, nuclear factor erythroid-derived-2-like 2 (Nrf2) is another cytoprotective signaling pathway that plays an important role in cellular antioxidative responses [23, 24]. Zhao et al. demonstrated that Akt-mediated Nrf2 activation via p62, an adaptor protein involved in autophagy, is associated with attenuation of type 1 diabetes-induced oxidative stress and apoptosis by resveratrol [25]. Additionally, they showed that upregulation of Nrf2 could prevent DCM in mice with type 2 diabetes [26]. It was reported that inhibition of sEH by stabilizing the levels of epoxyeicosatrienoic acids (EETs) upregulates Nrf2 levels and reduces oxidative and endoplasmic reticulum (ER) stress [27]. Additionally, 14, 15-EET was reported to protect against cigarette smoke condensate-induced lung inflammation by promoting the accumulation of Nrf2 in human bronchial epithelial cell [28]. Our previous study confirmed that EET prevents TNF-*α*-induced endothelial cell apoptosis by inhibiting the oxidative stress associated with Nrf2 activation [29]. However, whether Nrf2 activated by EETs plays a role in the prevention of DCM remains unclear.

Cytochrome P450 (CYP) epoxygenases convert arachidonic acid into four regioisomeric EETs (5, 6-EET, 8, 9-EET, 11, 12-EET, and 14, 15-EET) that perform diverse biological activities in the cardiovascular system [30]. sEH converts EETs to dihydroxyeicosatrienoic acids (DHETs) which has less active biologically [31]. Emerging evidence suggests that the inhibition of sEH is associated with important biological activities in various cardiovascular diseases. A proteomic analysis of the hearts from diabetic mice suggested that increased sEH expression and antioxidative effect are important changes in the early stages of DCM [32]. In addition, increasing EET levels through sEH inhibition prevents cardiac hypertrophy and dysfunction in the models of cardiac overload [33]. Recently, the CYP/sEH system has been demonstrated to be involved in autophagy and apoptosis. For example, 14, 15-EET protects cardiomyocytes during starvation by regulating autophagy [34]. The sEH inhibitor t-TUCB downregulates ER stress and increases hepatic autophagy in metabolic diseases [35]. Additionally, the pulmonary autophagy induced by cigarette smoke is reportedly attenuated in sEH-deficient mice [36]. In our previous study, we reported that 11, 12-EETs ameliorate the ethanol-induced cardiac dysfunction by increasing autophagy and inhibiting apoptosis [17]. However, the precise mechanisms by which sEH inhibition regulates autophagy and apoptosis in DCM remain unclear. Therefore, in the present study, we investigated the underlying mechanisms for the same using *in vivo* and *in vitro* models.

## 2. Methods

### 2.1. Generation of Recombinant Adeno-Associated Virus (AAV)

The rAAV-9 system given was from Dr. Xiao (University of North Carolina, Chapel Hill, NC, USA). Nrf2 siRNA oligos and scrambled sequences were designed and synthesized by Hanbio (Shanghai, China). AAV-Nrf2shRNA and AAV-ScshRNA were prepared via triple plasmid cotransfection of HEK293 cells, which is described previously [37].

### 2.2. Animals

All protocols involving animals were approved by the Institutional Animal Research Committee of Tongji Medical College, Huazhong University of Science and Technology (Wuhan, China). All animals received humane care in compliance with the guidelines of the National Institutes of Health (Bethesda, MD, USA) on the use of laboratory animals. C57BL/Ks background and 12-week-old male db/db mice and control db/m mice were purchased from Changzhou Cavens Laboratory Animal Co. Ltd. (Jiangsu, China) and were randomly assigned into the following groups (*n* = 8 per group): db/m+vehicle (Con), db/m+sEH inhibitor AUDA (AUDA), db/db+vehicle (DM), and db/db+sEH inhibitor AUDA (DM+AUDA). Next, we examined whether Nrf2 served as the downstream regulator of the sEH inhibitor AUDA. The shRNA of Nrf2 was used, and db/db mice and db/m mice were randomly allocated to the following groups (*n* = 8 in each group): db/m+vehicle+AAV-ScshRNA (ScshRNA+Con), db/db+vehicle+AAV-ScshRNA (ScshRNA+DM), db/m+vehicle+AAV-Nrf2shRNA (Nrf2shRNA+Con), db/m+sEH inhibitor AUDA+AAV-Nrf2shRNA (Nrf2shRNA+AUDA), db/db+vehicle+AAV-Nrf2shRNA (Nrf2shRNA+DM), and db/db+sEH inhibitor AUDA+AAV-Nrf2shRNA group (Nrf2shRNA+DM+AUDA). Fourteen-week-old mice were injected with AAV-Nrf2shRNA or AAV-ScshRNA (1 × 10^11^ PFU) through the tail vein, and at the age of 18 weeks, the mice were administered the sEH inhibitor AUDA (25 mg/L) through drinking water as previously described [38]. The rAAV-treated db/db mice and the control mice were sacrificed at 30 weeks, and tissue samples were snap-frozen in liquid nitrogen as well as collected for paraffin embedding.

### 2.3. Isolation of Primary Cardiomyocytes

The hearts from neonatal rats from 1- to 3-day-old were obtained, cut into small pieces, and digested with trypsin and type II collagenase at 37°C. The cells were filtered via a cell strainer (200 *μ*m mesh), planted into petri dishes, and incubated for 2 h. Cardiomyocytes were collected and cultured in low-glucose Dulbecco's Modified Eagle's Medium (DMEM), containing 10% fetal bovine serum, for further experiments. Specific isolated and cultured primary cardiomyocytes were prepared as described previously [39]. For HGHF treatment, the cells were cultured in DMEM containing 500 *μ*mol/L saturated free fatty acid palmitate and 25 mmol/L glucose for 24 h in the presence or absence of 1 *μ*M AUDA, as described previously [40]. To further determine the potential mechanisms of AUDA in regulating autophagy, the cells were pretreated with 10 nM bafilomycin A1, an autophagy and lysosome inhibitor, 0.5 h before the addition of AUDA to the culture medium.

### 2.4. Determination of Serum and Urine EETs and DHETs

Serum and urine samples were collected from all mice. ELISA kits (Detroit R&D, Detroit, MI, USA) were used to determine the concentrations of 11, 12-EET and its stable metabolite 11, 12-DHET, according to the manufacturer's instructions, as previously described [41].

### 2.5. Western Blot Analysis

Cardiac tissues and cardiomyocytes were extracted and homogenized. Protein concentration was measured using a bicinchoninic acid (BCA) kit (Boster, Wuhan China) according to the manufacturer's instructions, and western blotting was performed as described previously [39]. The following antibodies were used: Bax (BosterBio, Pleasanton, CA, USA), Bcl2 (Boster), caspase-3 (Proteintech, Rosemont, IL, USA), cleaved caspase-3 (Cell Signaling Technology (CST), Danvers, MA, USA), NOX2 (Proteintech), NOX4 (Proteintech), SOD1 (Proteintech), Beclin 1 (CST), Atg3 (Santa Cruz Biotechnology, Dallas, TX, USA), LC3II (CST), and *β*-actin (Santa Cruz Biotechnology).

### 2.6. Histological Analysis and Confocal or Immunofluorescence Microscopy

Heart sections were subjected to DHE, TUNEL, and 3-nitrotyrosine (3-NT) staining according to the manufacturer's instruction. The images were acquired using an inverted microscope (TE 2000, Nikon, Tokyo, Japan) equipped with a digital imaging camera. Additionally, sections were incubated with the primary antibody for 72 h at 4°C in a 0.1 N phosphate buffer, containing 1% Triton X-100 and 1% bovine serum albumin, and then visualized as described above [42].

### 2.7. Autophagic Flux Analysis

We selected H9c2 cells to observe autophagic flux owing to the poor activity and transfection efficacy of neonatal rat cardiomyocytes. Fluorescence microscopy-based detection of mCherry-GFP-LC3 (Vigenebio, Rockville, MD, USA) was performed according to the manufacturer's instructions, as previously described [43].

### 2.8. Gene Silencing

Lipofectamine 2000 (Invitrogen, Waltham, MA, USA) was used to transfect Nrf2 siRNA (Ruibo, Guangzhou, China) to cardiomyocytes according to the manufacturer's protocol. The efficiency of siRNA knockdown was determined via western blotting after 48 h of transfection.

### 2.9. Transmission Electron Microscopy (TEM)

Heart tissues (1 mm3) were fixed with 4% glutaraldehyde in a 0.1 M phosphate buffer at 4°C for 4 h. Next, the samples were postfixed with 1% osmium tetroxide at room temperature for 2 h. Then, they were embedded in araldite/812 after dehydration in graded ethanol (70–100%). Ultrathin sections were cut and stained with uranyl acetate and lead hydroxide and visualized using a Tecnai G2 12 transmission electron microscope (FEI, Hillsboro, OR, USA), as previously described [17].

### 2.10. Flow Cytometry Assay

We treated primary cardiomyocytes with or without Nrf2 knockdown, with HGHF in the presence or absence of AUDA. Then, the Annexin-V/PI apoptotic assay was used to analyze cardiomyocyte apoptosis according to the manufacturer's instructions, as described previously [9], via FACStar Plus flow cytometer (BD, Franklin Lakes, NJ, USA).

### 2.11. Nuclear-Cytosolic Fractionation

Cytosolic and nuclear fractions of cardiomyocytes were separated using the Subcellular Protein Fractionation Kit (Beyotime, Shanghai, China) according to the manufacturer's instructions.

### 2.12. RNA Extraction and Real-Time Polymerase Chain Reaction

Total RNA was extracted using TRIzol reagent (Invitrogen) according to the manufacturer's instructions. Next, cDNA was synthesized using EasyScript First-Strand cDNA Synthesis SuperMix (TransGen Biotech Corporation, Beijing, China). mRNA levels were quantified via qRT-PCR using Power SYBR Green PCR Master Mix (Invitrogen) with the primers listed in Supplemental Table 1. *β*-Actin was used as an internal control, and data were analyzed using the 2^-*ΔΔ*Ct^ method.

### 2.13. Statistical Analysis

All data are presented as mean ± standard error of mean (SEM). Statistical significance of differences among the groups was analyzed using Student's *t*-test or a one-way analysis of variance for multiple comparisons with Tukey's test which were run only when the *F*-test achieved a *p* value < 0.05, and there was no significant variance in homogeneity. All statistical analyses were performed using the SPSS 22.0 software (IBM, Armonk, NY, USA), and *p* < 0.05 was considered to represent a statistically significant difference.

## 3. Results

### 3.1. AUDA Administration Alleviated Cardiac Dysfunction and Reduced Apoptosis in Diabetic Mice Heart

**Figure 1:**
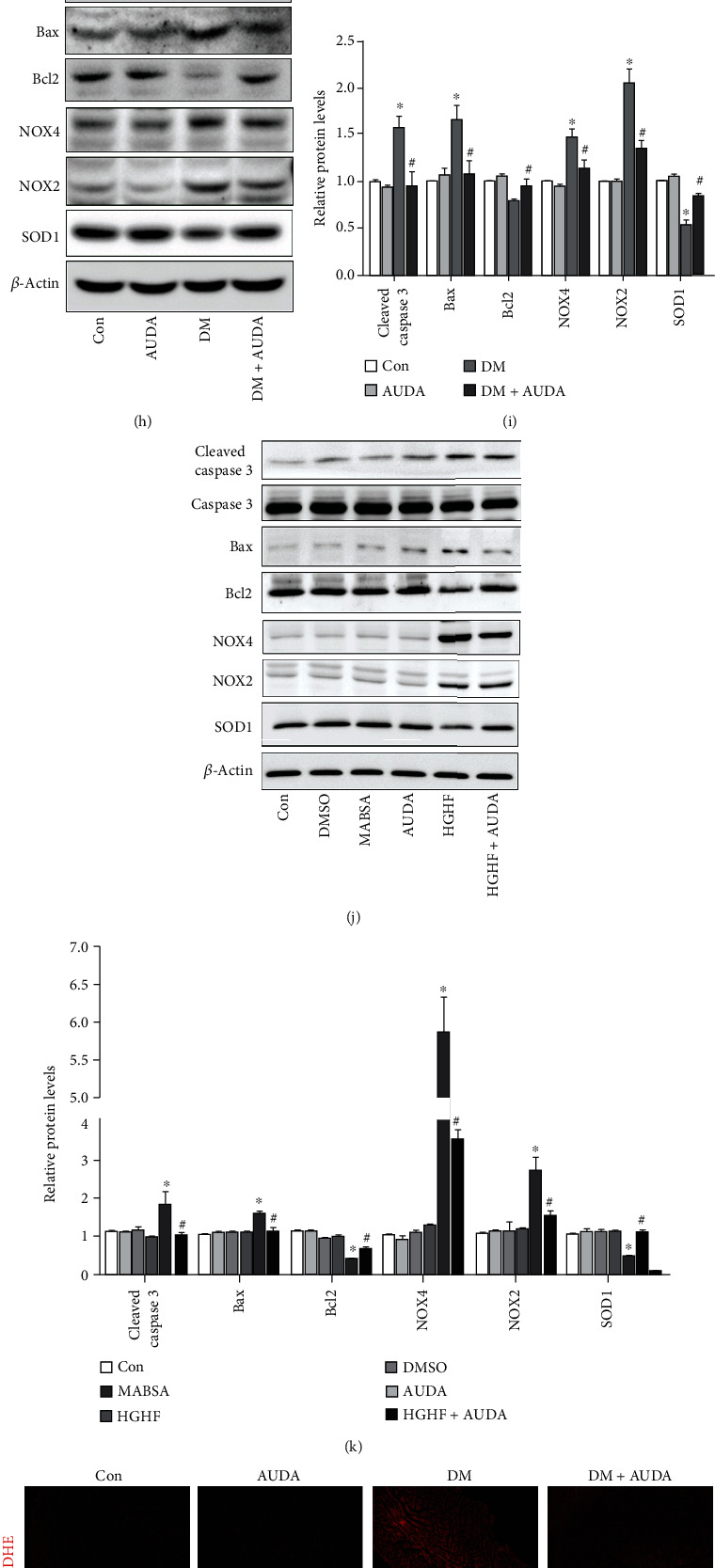
AUDA administration alleviated cardiac dysfunction and reduced apoptosis in db/db mice heart. (a) Representative immunoblots and quantitation of soluble epoxide hydrolase (sEH) expression, (b) +dp/dt max, (c) -dp/dt min, (d) left ventricular ejection fraction (LVEF), (e) left ventricular internal diameter end systole (LVID(s)), (f) left ventricular internal diameter end diastole (LVID(d)), and (g) E/A ratio. (h and i) Representative immunoblots and quantitation of protein expression of cleaved caspase-3, Bax, Bcl-2, NOX4, NOX2, and SOD1 in the myocardia of mice treated with or without the sEH inhibitor AUDA. (j and k) Representative immunoblots and quantitation of protein expression of cleaved caspase-3, Bax, Bcl-2, NOX4, NOX2, and SOD1 in HGHF-stimulated cardiomyocytes pretreated with or without AUDA. (l–o) Histological analysis and quantitation of DHE, TUNEL, and 3-NT staining in the myocardia in different groups. Data were expressed as mean ± SEM (*n* = 8 mice per group). ^∗^*p* < 0.05 vs. Con and ^#^*p* < 0.05 vs. DM or HGHF. Con: db/m+vehicle; AUDA: db/m+AUDA; DM: db/db+vehicle; MABSA: mannitol+ BSA; DHE: dihydroethidium; HGHF: high glucose and high fat.

To investigate the role of sEH in cardiac dysfunction, we detected sEH levels in the heart tissue of a DCM mouse model. As shown in Figure 1(a), sEH expressions were significantly increased in the db/db mice heart and were reduced by the sEH pharmacological inhibitor AUDA. Moreover, the administration of AUDA caused a significant increase in both the serum and urine levels of 11, 12-EET, while the levels of 11, 12-DHET decreased (Supplemental Fig. 1A–B). Besides, we also observed that 11, 12-EET levels in db/db mice were less than those in db/m mice (Supplemental Fig. 1A-B). sEH activity was calculated as the ratio of 11, 12-EET/DHET in the serum and urine, which showed that the inhibitor AUDA inhibited sEH activity (Supplemental Fig. 1C-D). The levels of markers of cardiac performance, dp/dt max, dp/dt min, and left ventricular ejection fraction (LVEF), were significantly increased in the AUDA-treated groups compared with the db/db group (Figures 1(b)–1(d)). Left ventricular internal diameter end systole (LVID(s)) and left ventricular internal diameter end diastole (LVID(d)) values were also reduced in the AUDA-treated groups (Figures 1(e) and 1(f)). Additionally, increased E/A ratios were noted in db/db mice (Figure 1(g)). The baseline parameters are shown in Supplemental Table 2. Immunoblots showed that the expression of proapoptotic proteins Bax and cleaved caspase-3 increased significantly in the hearts of diabetic mice, while the levels of the antiapoptotic protein Bcl-2 decreased (Figures 1(h) and 1(i)). In addition, the NOX2 and NOX4 expressions were significantly increased in the diabetic myocardium, whereas the SOD1 expression was downregulated. However, treatment with the sEH inhibitor AUDA reversed these effects (Figures 1(h) and 1(i)). Moreover, we observed similar effects in the HGHF-induced neonatal cardiomyocytes (Figures 1(j) and 1(k)). These data was consistent with the results from DHE, TUNEL, and 3-NT staining (Figures 1(l)–1(o)). Additionally, flow cytometry revealed that the sEH inhibitor AUDA inhibited HGHF-induced apoptosis in neonatal cardiomyocytes (Supplemental Fig. 2A-B). Immunofluorescence analysis also showed reduced ROS levels in cardiomyocytes after HGHF administration (Supplemental Fig. 2C-D). These data demonstrated that the sEH inhibitor AUDA reduced apoptosis and oxidative stress and reversed cardiac dysfunction in the diabetic myocardium.

### 3.2. AUDA Administration Increased Autophagy in Diabetic Mice Heart and Promoted Autophagic Flux in HGHF-Treated Cardiomyocytes *In Vitro*

**Figure 2:**
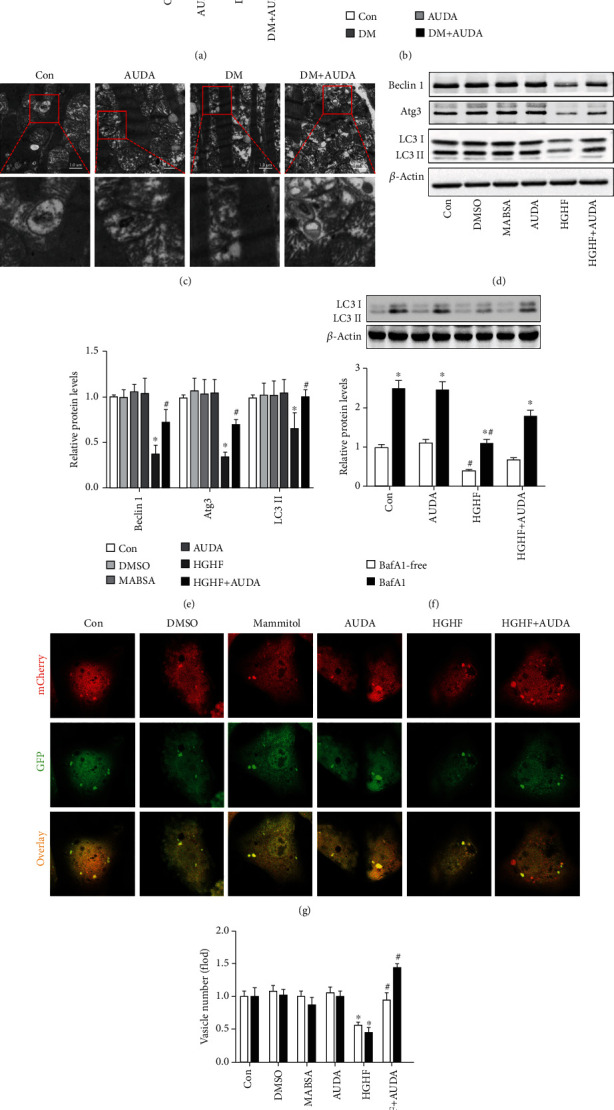
AUDA administration increased autophagy in diabetic mice heart and promoted autophagic flux under HGHF-treated cardiomyocytes *in vitro.* (a and b) Representative immunoblots and quantitation of protein level of Beclin 1, Atg3, and LC3II in the myocardia of mice from different groups. (c) Representative autophagic vacuoles from cardiac tissues visualized via transmission electron microscopy. (d and e) Representative immunoblots and quantitation of protein level of Beclin 1, Atg3, and LC3II in HGHF-stimulated cardiomyocytes in different groups. (f) LC3-II/*β*-actin ratio with or without bafilomycin A1 treatment during culture in HGHF conditions. (g and h) Representative images of fluorescent LC3 puncta and quantitation of cardiomyocytes with different treatments. Data were expressed as mean ± SEM (n ≥ 3 per group). ^∗^p < 0.05 vs. Con or BafA1-free and ^#^p < 0.05 vs. DM or HGHF. Con: db/m+vehicle or control; AUDA: db/m+AUDA or AUDA; DM: db/db+vehicle; MABSA: mannitol+BSA; HGHF: high glucose and high fat.

Western blotting results showed that the autophagic markers Beclin-1, Atg3, and LC3 II were significantly downregulated in the heart tissues of diabetic mice, and the expression was reversed with the sEH inhibitor AUDA (Figures 2(a) and 2(b)). Electron microscopic images showed fewer autophagosomes in the heart tissues of db/db mice than in db/m mice, whereas the administration of AUDA upregulated autophagy in diabetic mice hearts (Figure 2(c)). To clarify the pathogenesis of DCM *in vitro* and to explore the effects of AUDA on autophagy, we used neonatal cardiomyocytes. Immunoblots showed that AUDA significantly reversed the HGHF-induced downregulation of Beclin-1, Atg3, and LC3 II in neonatal cardiomyocytes (Figures 2(d) and 2(e)). Interestingly, we found that the administration of AUDA increased autophagic flux, as evidenced by an increased LC3-II/*β*-actin ratio under bafilomycin A1 treatment, a lysosomal inhibitor used to evaluate autophagic flux (Figure 2(f)). To separately evaluate the extent of autophagosome and autolysosome formation, we used an adenovirus harboring tandem fluorescent mCherry-GFP-LC3 to detect autophagic flux. GFP loses fluorescence in the acidic environment of lysosomes, whereas mCherry retains fluorescence in the same conditions. Thus, merged yellow LC3 puncta indicate autophagosomes, whereas red LC3 puncta indicate autolysosomes, thereby allowing us to detect the autophagic flux. Figures 2(g) and 2(h) showed that AUDA increased the HGHF-induced downregulation of yellow and red vesicles. Collectively, these data confirmed that AUDA increased autophagy in the hearts of diabetic mice and in HGHF-treated cardiomyocytes.

### 3.3. AUDA Administration Upregulated Nrf2 Level and Promoted Its Nuclear Translocation in Diabetic Cardiomyocytes Relying on p-Akt

**Figure 3:**
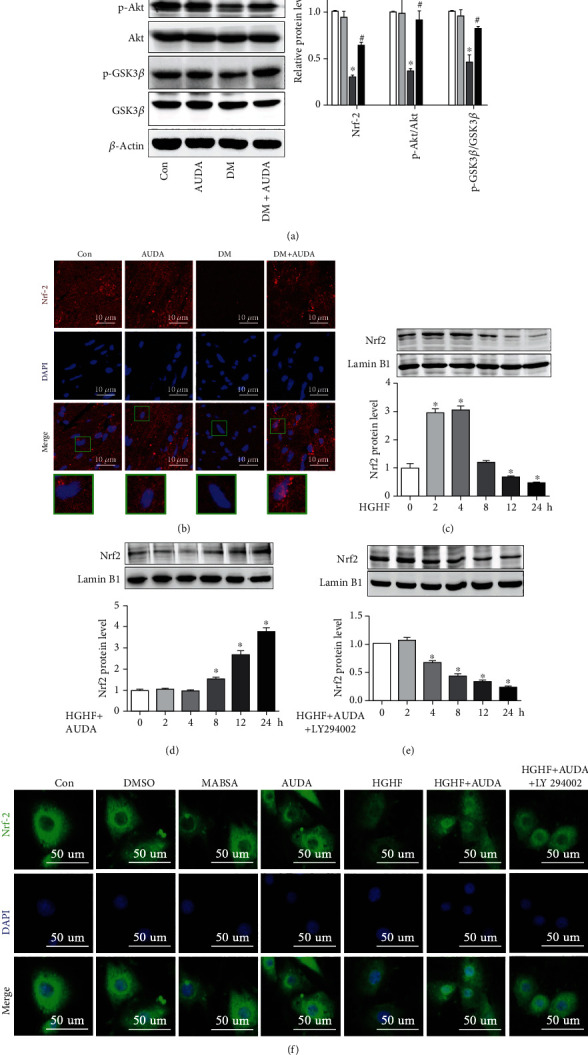
AUDA administration upregulated the expression of Nrf2 and promoted its nuclear translocation rely on p-Akt. (a) Representative immunoblots and quantitation of protein expression of Nrf2, p-Akt, Akt, p-GSK3*β*, and GSK3*β* in the myocardia of mice from different groups. (b) Confocal observation of immunofluorescent staining for Nrf2 in the myocardia of mice from different groups. (c) Representative immunoblots of nuclear Nrf2 expression in HGHF-stimulated cardiomyocytes at different time points. (d) Representative immunoblots of nuclear Nrf2 expression in the HGHF-stimulated cardiomyocytes pretreated with AUDA at different time points. (e) Representative immunoblots of nuclear Nrf2 expression in the HGHF- and AUDA-stimulated cardiomyocytes pretreated with 50 *μ*M LY294002 at different time points. (f) Representative images of fluorescent Nrf2 expression in the HGHF-stimulated cardiomyocytes in different groups. Data were expressed as mean ± SEM (n ≥ 3 per group). ^∗^p < 0.05 vs. Con or 0 h and ^#^p < 0.05 vs. DM. Con: db/m+vehicle or control; AUDA: db/m+AUDA or AUDA; DM: db/db+vehicle; MABSA: mannitol+BSA; HGHF: high glucose and high fat.

Various studies have shown that Nrf2 plays a key role in DCM development [20, 44, 45]. Moreover, Nrf2 has been shown to be upregulated by EET in cigarette smoke condensate-induced inflammation in lung epithelial cells [28]. In the present study, we investigated whether the sEH inhibitor AUDA protects the heart from diabetes by activating Nrf2, by analyzing the expression of Nrf2 and its related regulatory molecules in the hearts of diabetic mice. Immunoblotting results demonstrated that the protein level of Nrf2, p-Akt, and p-GSK3*β* was significantly reduced in diabetic hearts; however, this effect was reversed by administration of AUDA (Figure 3(a)). Additionally, the ratio of nuclear Nrf2 to cytosolic Nrf2 was reduced in db/db mice and increased following the administration of AUDA, suggesting that the transcriptional activity of Nrf2 was increased (Figure 3(b)).  Figure 3(c) shows that the nuclear Nrf2 expression in cardiomyocytes increased after 2 h of HGHF stimulation but significantly decreased after 12 h of HGHF stimulation. We also observed that the sEH inhibitor AUDA reversed these effects in a time-dependent manner (Figure 3(d)). Furthermore, we evaluated the effect of LY294002, a PI3K inhibitor, to influence Nrf2 expression. Immunoblotting results demonstrated that the addition of 50 *μ*M LY294002 reduced Nrf2 expression in the nucleus (Figure 3(e)). Similar results were observed using immunofluorescence staining (Figure 3(f)). Overall, these results suggest that the sEH inhibitor AUDA might exert its protective effects by regulating Nrf2 function through p-Akt.

### 3.4. AUDA Administration Regulated Autophagy and Apoptosis in Diabetic Cardiomyocytes Relied on Nrf2

**Figure 4:**
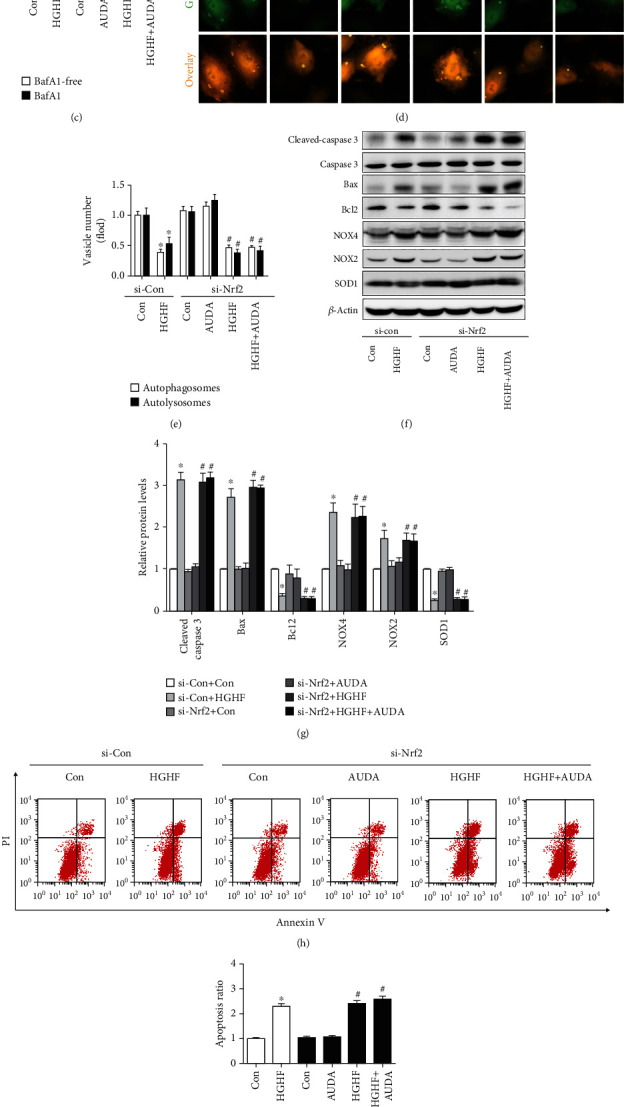
Small interfering RNA of Nrf2 specifically reduced the protective effects of AUDA on cardiomyocytes autophagy and apoptosis. (a and b) Representative immunoblots and quantitation of protein expression of Beclin 1, Atg3, and LC3II in HGHF-stimulated cardiomyocytes in different groups. (c) LC3-II/*β*-actin ratio with or without bafilomycin A1 treatment under HGHF culture conditions. (d and e) Representative images of fluorescent LC3 puncta and quantitation of cardiomyocytes with different treatments. (f and g) Representative immunoblots and quantitation of protein expression of cleaved caspase-3, Bax, Bcl-2, NOX4, NOX2, and SOD1 in the HGHF-stimulated cardiomyocytes pretreated with or without AUDA. (h and i) Representative images and quantitation of cardiomyocyte apoptosis via the Annexin-V/PI apoptotic assay. (j and k) Representative images and quantitation of cardiomyocyte ROS levels via DCFH-DA. Data were expressed as mean ± SEM (n ≥ 3 per group). ^∗^p < 0.05 vs. si-Con+Con and ^#^p < 0.05 vs. si-Nrf2+Con. HGHF: high glucose and high fat.

To explore the role of Nrf2 regulating diabetic cardiomyocyte autophagy and apoptosis, we knocked down Nrf2 by transfecting neonatal cardiomyocytes with an Nrf2-specific siRNA (si-Nrf2). Supplemental Fig. 3A shows that Nrf2 knockdown with si-Nrf2 was effective. Immunoblots demonstrated that the expression of Beclin 1, Atg3, and LC3-II was not changed upon AUDA administration when Nrf2 was knocked down under HGHF treatment (Figures 4(a) and 4(b)). In addition, AUDA failed to increase autophagic flux in the Nrf2 knockdown cardiomyocytes stimulated by HGHF (Figures 4(c)–4(e)). We also observed that Bax, cleaved caspase-3, NOX4, and NOX2 levels were upregulated and Bcl-2 and SOD1 levels were downregulated in HGHF-induced neonatal cardiomyocytes following treatment with AUDA, but these effects were similar to the untreated group (Figures 4(f) and 4(g)). Moreover, flow cytometry and immunofluorescence results were consistent with the above-mentioned results (Figures 4(h)–4(k)). These results demonstrated that the sEH inhibitor AUDA promoted cardiac autophagy and prevented cardiomyocyte apoptosis via Nrf2.

### 3.5. Virus Carrying Code for Nrf2 shRNA Specifically Reduced the Protective Effects of AUDA on Cardiac Function and Autophagy in db/db Mice

**Figure 5:**
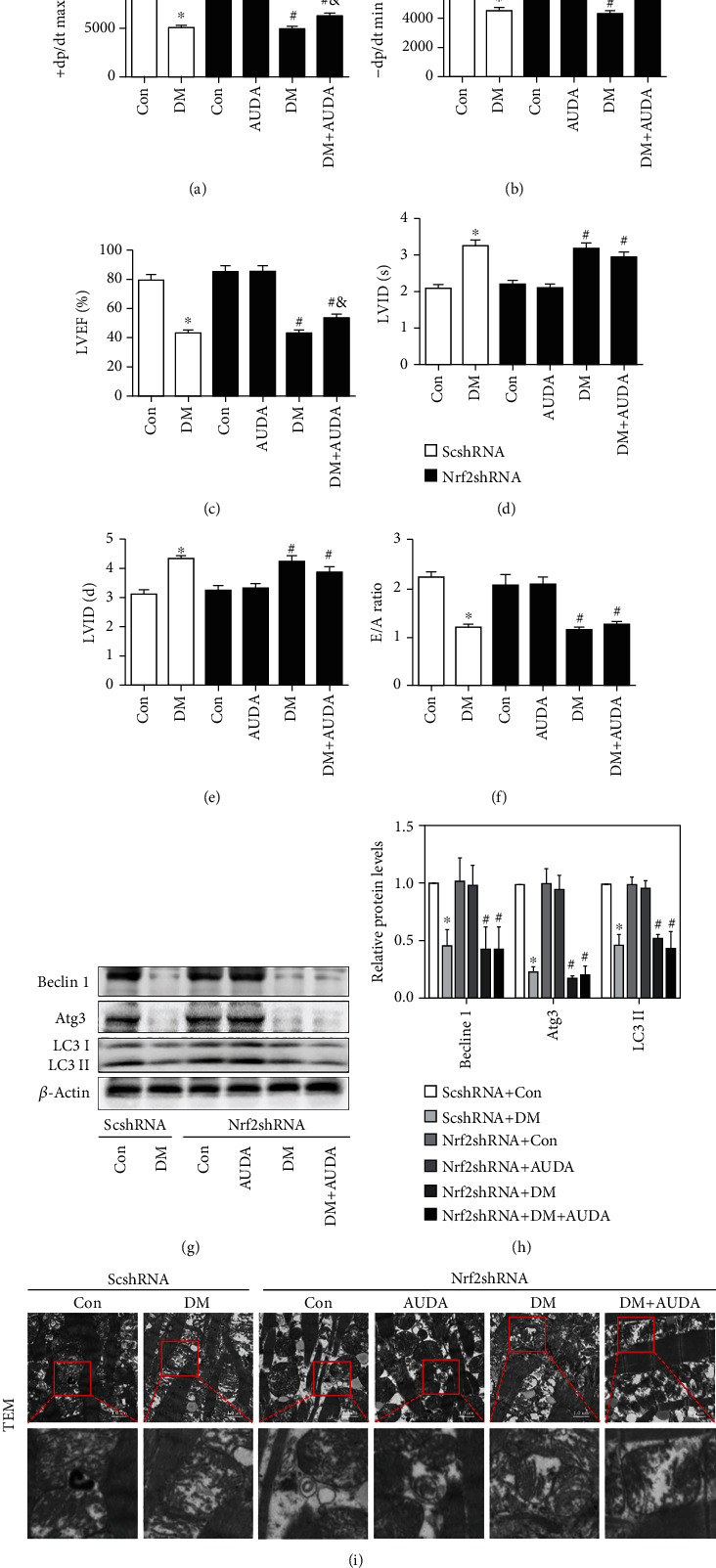
Virus carrying code for Nrf2 shRNA specifically reduced the protective effects of AUDA on cardiac function and autophagy level in db/db mice. (a) +dp/dt max, (b) -dp/dt min, (c) LVEF, (d) LVID(s), (e) LVID(d), and (f) E/A ratio. (g and h) Representative immunoblots and quantitation of protein expression of Beclin 1, Atg3, and LC3II in the myocardia of mice from different groups. (i) Representative images of autophagic vacuoles in cardiac tissues by transmission electron microscopy. Data were expressed as mean ± SEM (n = 8 per group). ^∗^p < 0.05 vs. ScshRNA+Con, ^#^p < 0.05 vs. Nrf2shRNA+Con, and ^&^p < 0.05 vs. Nrf2shRNA+DM. ScshRNA: AAV-ScshRNA; Nrf2shRNA: AAV-Nrf2shRNA; Con: db/m+vehicle; AUDA: db/m+AUDA; DM: db/db+vehicle.

To explore the role of Nrf2 in the regulation of cardiac function in diabetic mice, we used shRNA to knockdown the Nrf2 gene in mice. The mRNA and protein levels of Nrf2 were significantly reduced after treatment with AAV-Nrf2-shRNA for four months (Supplemental Fig. 3B-C). The administration of the sEH inhibitor AUDA caused a significant increase in both the serum and urine levels of 11, 12-EET, while the level of 11, 12-DHET declined (Supplemental Fig. 4A-B). sEH activity in the serum and urine was analyzed as the ratio of 11, 12-EET/DHET, which showed that sEH activity was inhibited by the sEH inhibitor AUDA. Moreover, Nrf2 silencing had no effect on sEH activity and 11, 12-EET levels (Supplemental Fig. 4C-D). Additionally, dp/dt max, dp/dt min, and LVEF were significantly reduced in AUDA-treated mice after Nrf2 knockdown (Figures 5(a)–5(c)). LVID(s) and LVID(d) also increased in the AUDA/Nrf2-shRNA-treated db/db group (Figures 5(d) and 5(e)). The administration of the sEH inhibitor AUDA in Nrf2 knockdown diabetic mice did not increase the E/A ratio (Figure 5(f)). The baseline parameters are shown in Supplemental Table 3. Immunoblots showed that AUDA did not upregulate the autophagic markers Beclin-1, Atg3, and LC3 II/I protein levels (Figures 5(g) and 5(h)). Transmission electron microscopy revealed that the number of autophagosomes in the db/db+AUDA group was the same as in the control (Figure 5(i)). These data show that knockdown of Nrf2 abolished the effect of AUDA on autophagy in diabetic mice heart.

### 3.6. Virus Carrying Code for Nrf2 shRNA Partially Reduced the Protective Effects of AUDA on Apoptosis in Db/Db Mice

**Figure 6:**
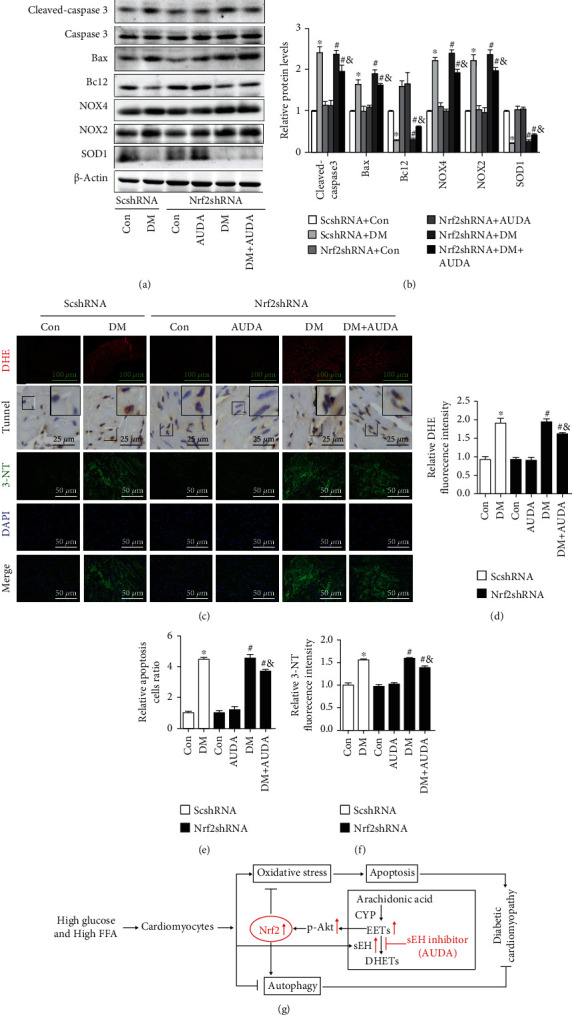
Virus carrying code for Nrf2 shRNA specifically reduced the protective effects of AUDA on apoptosis in db/db mice heart. (a and b) Representative immunoblots and quantitation of protein expression of cleaved caspase-3, Bax, Bcl-2, NOX4, NOX2, and SOD1 in the myocardia of mice from different groups. (c–f) Histological analysis and quantitation of DHE, TUNEL, and 3-NT staining in the myocardia from different groups. (g) Mechanism diagram of full text. Data were expressed as mean ± SEM (n = 8 per group). ^∗^p < 0.05 vs. ScshRNA +Con, ^#^p < 0.05 vs. Nrf2shRNA+Con, and ^&^p < 0.05 vs. Nrf2shRNA+DM. ScshRNA: AAV-ScshRNA; Nrf2shRNA: AAV-Nrf2shRNA; Con: db/m+vehicle; AUDA: db/m+AUDA; DM: db/db+vehicle; DHE: dihydroethidium.

In this study, we observed that AUDA attenuated the HGHF-induced cardiomyocyte apoptosis. However, it was reversed after knockdown Nrf2. Additionally, we analyzed the effect of Nrf2 on cardiomyocyte apoptosis in db/db mice following the administration of AUDA. Immunoblots showed that AUDA partially reduced expression of the Bax and cleaved caspase-3 and increased Bcl-2 expression compared to that group not treated with Nrf2shRNA, which was different to the results observed *in vitro* (Figures 6(a) and 6(b)). Moreover, NOX2 and NOX4 protein levels were also partially reduced, and the expression of SOD1 was partially increased following treatment with AUDA, compared to that in the group not treated with AAV-Nrf2shRNA (Figures 6(a) and 6(b)). The same results were observed with DHE, TUNEL, and 3-NT staining (Figures 6(c)–6(f)). These data demonstrated that Nrf2 knockdown partially reversed the antiapoptotic effect of AUDA *in vivo*.

## 4. Discussion

In the present study, effects of the sEH inhibitor AUDA on DCM were investigated both *in vivo* and *in vitro*. Our results suggest that the administration of AUDA significantly prevented cardiac dysfunction in diabetic mice. In addition, we provide evidence that AUDA exerts beneficial effects by increasing autophagy and reducing apoptosis in diabetic heart. These data demonstrate that sEH inhibition may be a novel approach for DCM treatment.

sEH converts EETs into biologically inactive DHETs. In this study, we observed that sEH protein expression was significantly higher in the hearts of diabetic mice, suggesting that sEH inhibition might be a potential therapeutic target in cardiovascular diseases. AUDA is a recently synthesized, stable, and potent sEH inhibitor. AUDA also has an N-carboxylic acid substitution which increases water solubility without an appreciable reduction in the potency of sEH inhibition [46]. Consistent with our expectations, treatment with AUDA inhibited sEH enzyme activity and decreased sEH protein expression. It increased the serum levels of EETs in mice. In this study, we found that sEH inhibition significantly attenuated cardiac dysfunction, induced autophagy, and reduced cardiomyocyte apoptosis in db/db mice. This was also observed *in vitro*. Our results were consistent with those of previous studies showing that AUDA attenuates myocardial ischemia-reperfusion injury [47], prevents apoptosis [48, 49], and increases autophagy [34, 35]. Overall, these results demonstrate that sEH inhibition exerts beneficial effects against DCM.

Autophagy promotes cell survival by degradation of intracellular components such as long-lived or damaged proteins and organelles [50]. There are some controversies in some studies, and most studies have reported that insufficient autophagy is involved in DCM [51, 52], while others have shown opposite results [53]. The complicated findings may be due to a number of reasons. First, upregulation of autophagy has been accounted for in the pathogenesis of DCM in the type 1 diabetic mice heart [54]. Second, the pathogenesis of diabetes is very complex. Apart from insulin deficiency or insulin resistance, diabetes is often accompanied by varying degrees of hyperglycemia, dyslipidemia, and other signaling changes which can also affect autophagy. High glucose levels directly suppress autophagy in cardiomyocytes [55], and high levels of palmitic acid can also inhibit autophagy [56]. Therefore, the ultimate functional status of autophagy in the heart of diabetic mouse reflects the net effect of insulin resistance or deficiency, hyperglycemia, dyslipidemia, or other changes associated with diabetes. Third, cardiac autophagy is suppressed in metabolic syndrome induced by a high-fat diet [51, 57, 58], while it is activated by fructose-induced hyperglycemia and insulin resistance [59]. Moreover, a milk fat-based diet also activates autophagy in mice heart [60]. The different cardiac autophagic responses can be partially explained by the variation in the duration of dietary intervention and the types and relative contents of diets [61]. Lastly, not all researches have analyzed autophagic flux in the heart, which may have contributed to the different opinion. In this study, we found that myocardial autophagy was decreased, as evidenced by reduced expression of Beclin1, Atg3, and LC3-II in the diabetic mice heart, which was reversed by the sEH inhibitor AUDA. In addition, we observed that AUDA enhanced autophagic flux, which evidenced by the enhanced LC3-II/*β*-actin ratio in the treatment of the lysosomal inhibitor bafilomycin A1. However, Nrf2 knockdown failed to show these effects despite administration of AUDA. These results suggest that the sEH inhibitor AUDA may hinder the development of DCM by upregulating cardiomyocyte autophagy via Nrf2 upregulation and transcriptional activation of Nrf2.

Both autophagy and apoptosis are involved in DCM pathogenesis [62]. Accumulating evidence suggests that the antiapoptotic effects of sEH inhibition protect the cells against cardiomyocyte injury [63, 64]. Consistent with these data, our results showed that the induction of apoptosis increased in diabetic mice and the cardiomyocytes stimulated by HGHF, and these effects were reversed upon the administration of AUDA. These effects lead to a significant reduction in cardiac dysfunction, which further confirmed the key role of sEH inhibition in cardiomyocyte apoptosis as a protective strategy against DCM. Furthermore, we also observed excessive myocardial ROS production, decreased antioxidant activity, and increased protein expression of NADPH oxidase subunits in DCM, whereas sEH inhibition overtly reversed these effects. Importantly, we found that attenuation of oxidative stress and apoptosis by the sEH inhibitor AUDA in DCM partially relied on Nrf2 activation.

Evidences show that both autophagy and apoptosis are tightly regulated by Nrf2 [65, 66]. In addition, Nrf2 plays a pivotal role in the development of DCM. However, whether Nrf2 is involved in the sEH inhibition-induced protection against DCM remains unclear. In this study, we found that AUDA induced the upregulation and transcriptional activation of Nrf2 relying on p-Akt. Importantly, Nrf2 knockdown abrogated the effects of AUDA on cardiomyocyte autophagy and reduced its effects on cardiac function and cardiomyocyte apoptosis in DCM. This may be because, in addition to Nrf2, many other transcription factors or cell types are also involved in AUDA-regulated DCM. These results strongly support the involvement of Nrf2 signaling pathway in mediating the beneficial effects of AUDA against DCM.

## 5. Conclusion

Our study demonstrated that the sEH inhibitor AUDA increased autophagy, decreased apoptosis, and alleviated cardiac dysfunction during DCM development. We also demonstrated that the underlying mechanisms of AUDA are associated with the upregulation of Nrf2 expression and promotion of its nuclear translocation (Figure 6(g)). Our study provides important insights into DCM pathogenesis, which is essential for the development of new therapeutic strategies.

### 5.1. Perspectives

Despite growing evidence demonstrating the important role of the CYP/sEH system in a variety of cardiovascular diseases, its role in DCM remains poorly understood. This study clarifies the mechanism of the sEH inhibitor AUDA in the regulation of autophagy and apoptosis in DCM and provides novel insights into new therapeutic strategies for DCM mediated by sEH inhibition. Our study represents an important step in understanding the roles and underlying mechanisms of the CYP/sEH system in DCM and provides potential new strategies for the treatment of DCM.

## Data Availability

The data used to support the findings of this study are available from the corresponding authors upon request.

## Supplementary Materials

Supplementary 1 Supplemental Table 1: primer of q RT-PCR. Supplemental Table 2. the baseline parameters about mice in different group. Supplemental Table 3. the baseline parameters about mice in different groups.

Supplementary 2 Supplemental Fig. 1: sEH inhibitor AUDA increased circulating EETs level in mice. Supplemental Fig. 2: AUDA administration reduced apoptosis and oxidative stress in cardiomyocytes. Supplemental Fig. 3: small interfering RNA of Nrf2 and virus carrying code for Nrf2 shRNA worked in cardiomyocytes and mice heart. Supplemental Fig. 4: sEH inhibitor AUDA increased circulating EETs level in mice.
