# Supplementary material for: Soluble Epoxide Hydrolase Inhibition Protected against Diabetic Cardiomyopathy through Inducing Autophagy and Reducing Apoptosis Relying on Nrf2 Upregulation and Transcription Activation
Source: Oxid Med Cell Longev. 2022 Mar 25;2022:3773415. doi: 10.1155/2022/3773415 (PMC8976467; doi:10.1155/2022/3773415)
Supplement: Supplementary 1 — Supplemental Table 1: primer of q RT-PCR. Supplemental Table 2. the baseline parameters about mice in different group. Supplemental Table 3. the baseline parameters about mice in different groups. [file 3773415.f1.doc]

**Supplement Table**

**Supplemental** Table 1. Primer of q RT-PCR

|  | Primer sequence |
| --- | --- |
| Nrf2-F | 5' CTACTCCCAGGTTGCCCAC 3' |
| Nrf2-R | 5' AGCTGCTTGTTTTCGGTATTAA 3' |
| β-actin-F | 5' AGAAGCTGTGCTATGTTGCTCTA3' |
| β-actin-R | 5' TCAGGCAGCTCATAGCTCTTC3' |

**Supplemental Table 2. Basic parameters of mice**

| Parameter | Con | AUDA | DM | DM+AUDA |
| --- | --- | --- | --- | --- |
| Heart/Body weight (g/kg) | 4.45±0.11 | 4.63±0.16 | 2.85±0.14^*^ | 2.73±0.20^*^ |
| Heart rate (bpm) | 517±24 | 524±24 | 467±17^*^ | 487±19^*^ |
| SBP (mmHg) | 113±4 | 102±4 | 113±3 | 101±3 |
| DBP (mmHg) | 78±5 | 70±3 | 72±4 | 69±4 |
| Blood glucose (mmol/l) | 6.98±1.61 | 7.53±0.76 | 23.34±2.09^*^ | 15.12±3.60^*#^ |

Data are expressed as Mean±SEM, n = 8 mice per group, *p<0.05 vs Con, #p <0.05 vs DM.

Con: db/m+vehicle group; AUDA: db/m+AUDA group; DM: db/db+vehicle group.

**Supplemental Table 3. Basic parameters of mice**

|  | ScshRNA | | | Nrf2shRNA | | | | | | | |  | |
| --- | --- | --- | --- | --- | --- | --- | --- | --- | --- | --- | --- | --- | --- |
| Parameter | Con | DM | | Con | | AUDA | | DM | | | DM+AUDA |  | |
| Heart/Body weight (g/kg) | 4.55±0.07 | | 2.91±0.12^*^ | | 4.33±0.10 | | 4.17±0.11 | | 2.71±0.12^#^ | 2.98±0.10^#^ | | | |
| Heart rate (bpm) | 496±27 | | 426±17^*^ | | 480±8 | | 478±10 | | 438±18^#^ | 441±15^#^ | | |  |
| SBP (mmHg) | 111±2 | | 118±3 | | 115±3 | | 102±5 | | 117±7 | 101±3 | | |  |
| DBP (mmHg) | 79± 3 | | 81±4 | | 83±1 | | 77±2 | | 81±6 | 77±6 | | |  |
| Blood glucose (mmol/l) | 6.39±3.13 | | 20.77±1.57^*^ | | 7.03±0.87 | | 6.40±3.54 | | 24.58±2.25^#^ | 16.03±1.50^#&^ | | |  |

Data are expressed as Mean±SEM, n = 8 mice per group, *p<0.05 vs ScshRNA+Con, #p <0.05 vs Nrf2shRNA+Con, &p <0.05 vs Nrf2shRNA+DM. ScshRNA: AAV-ScshRNA; Nrf2shRNA: AAV-Nrf2shRNA; Con: db/m+vehicle group; AUDA: db/m+AUDA group; DM: db/db+vehicle group
