# Supplementary material for: Soluble Epoxide Hydrolase Inhibition Protected against Diabetic Cardiomyopathy through Inducing Autophagy and Reducing Apoptosis Relying on Nrf2 Upregulation and Transcription Activation
Source: Oxid Med Cell Longev. 2022 Mar 25;2022:3773415. doi: 10.1155/2022/3773415 (PMC8976467; doi:10.1155/2022/3773415)
Supplement: Supplementary 2 — Supplemental Fig. 1: sEH inhibitor AUDA increased circulating EETs level in mice. Supplemental Fig. 2: AUDA administration reduced apoptosis and oxidative stress in cardiomyocytes. Supplemental Fig. 3: small interfering RNA of Nrf2 and virus carrying code for Nrf2 shRNA worked in cardiomyocytes and mice heart. Supplemental Fig. 4: sEH inhibitor AUDA increased circulating EETs level in mice. [file 3773415.f2.doc]

**Supplement Information**

Supplemental Figure 1


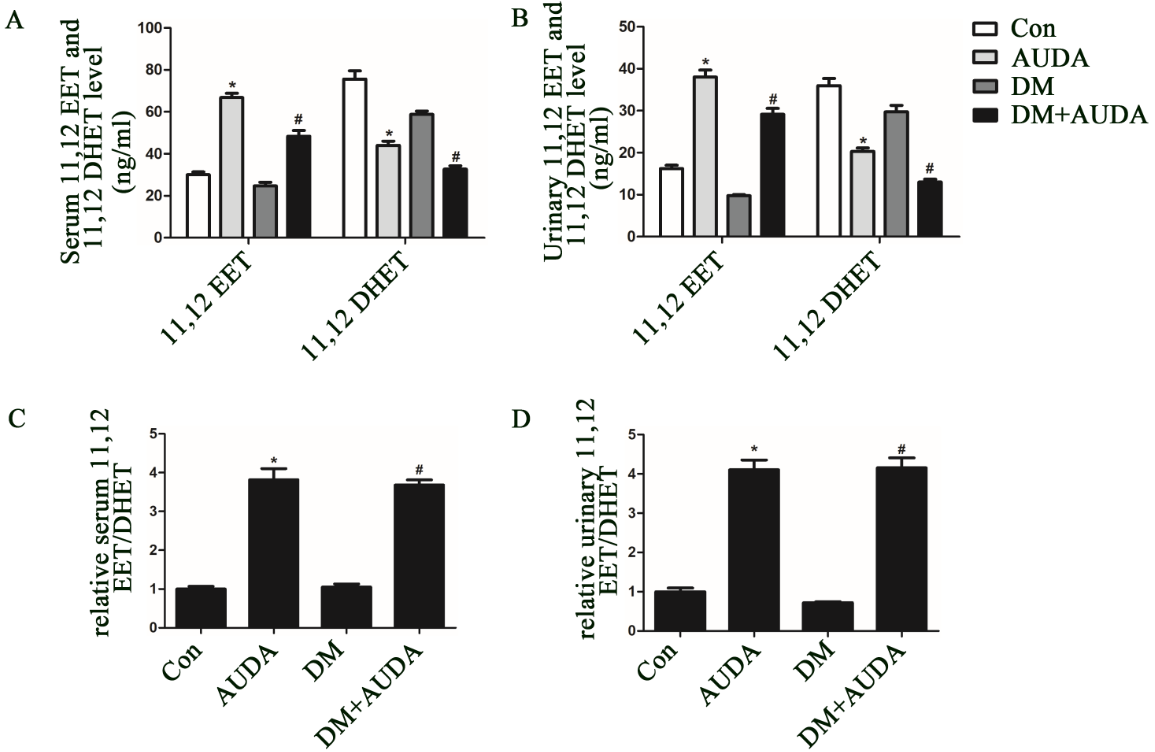


**Supplemental Figure 1. sEH inhibitor AUDA increased circulating EETs level in mice.** The concentration of 11, 12-EET and the corresponding 11, 12-DHET in serum (A) and urine (B) of mice which treated with AUDA. 11, 12 EET/DHET in serum (C) and urine (D) were calculated as index of sEH activity. Data were expressed as Mean ± SEM, n=8 per group, *p < 0.05 vs Con, #p < 0.05 vs DM. Con: db/m+vehicle group; AUDA: db/m+AUDA group; DM: db/db+vehicle group.

Supplemental Figure 2


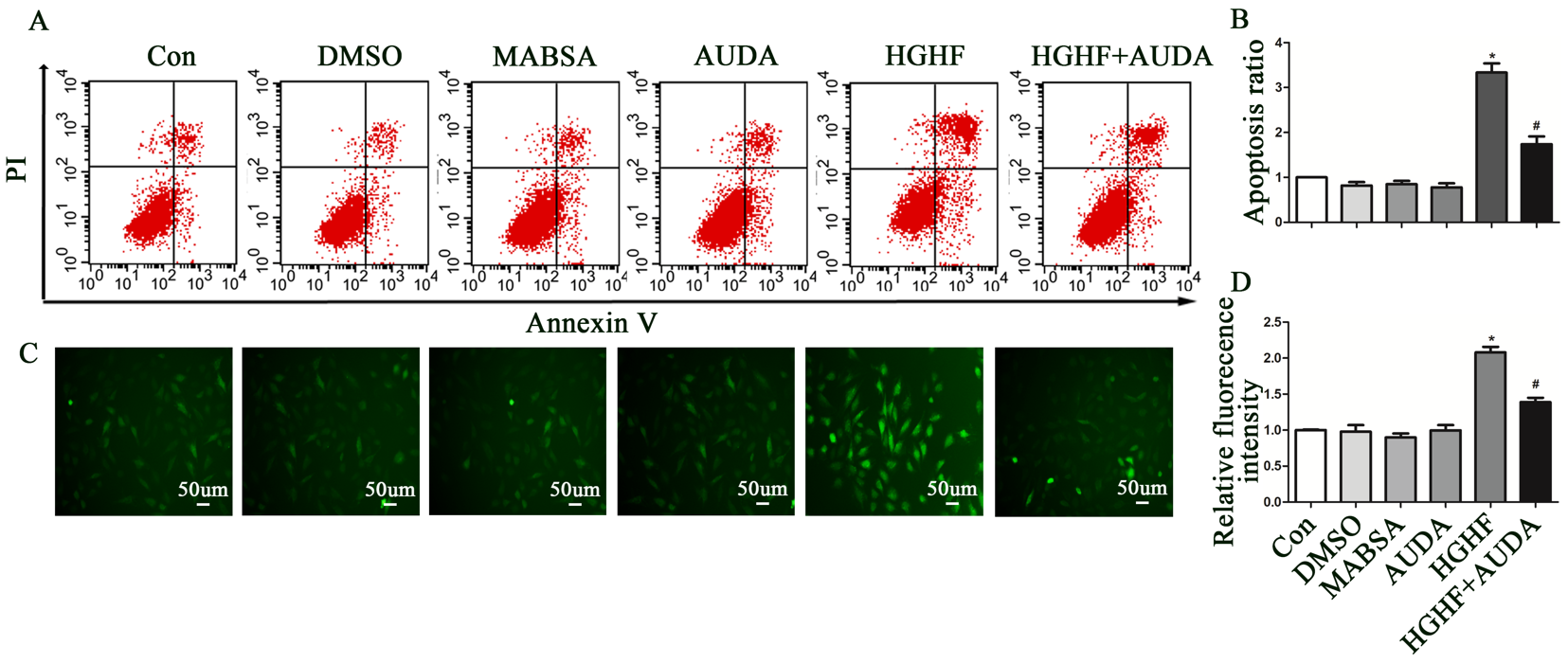


**Supplemental Figure 2. AUDA administration reduced apoptosis and oxidative stress in cardiomyocytes.** (A-B) Representative images and quantitation of cardiomyocyte apoptosis measured by Annexin-V/PI apoptotic assay. (C-D) Measurement of ROS levels in cardiomyocytes by DCFH-DA. Data were expressed as Mean ± SEM, n≥3 per group, *p < 0.05 vs Con, #p < 0.05 vs HGHF. MABSA: mannitol+ BSA; HGHF: high glucose and fat.

Supplemental Figure 3


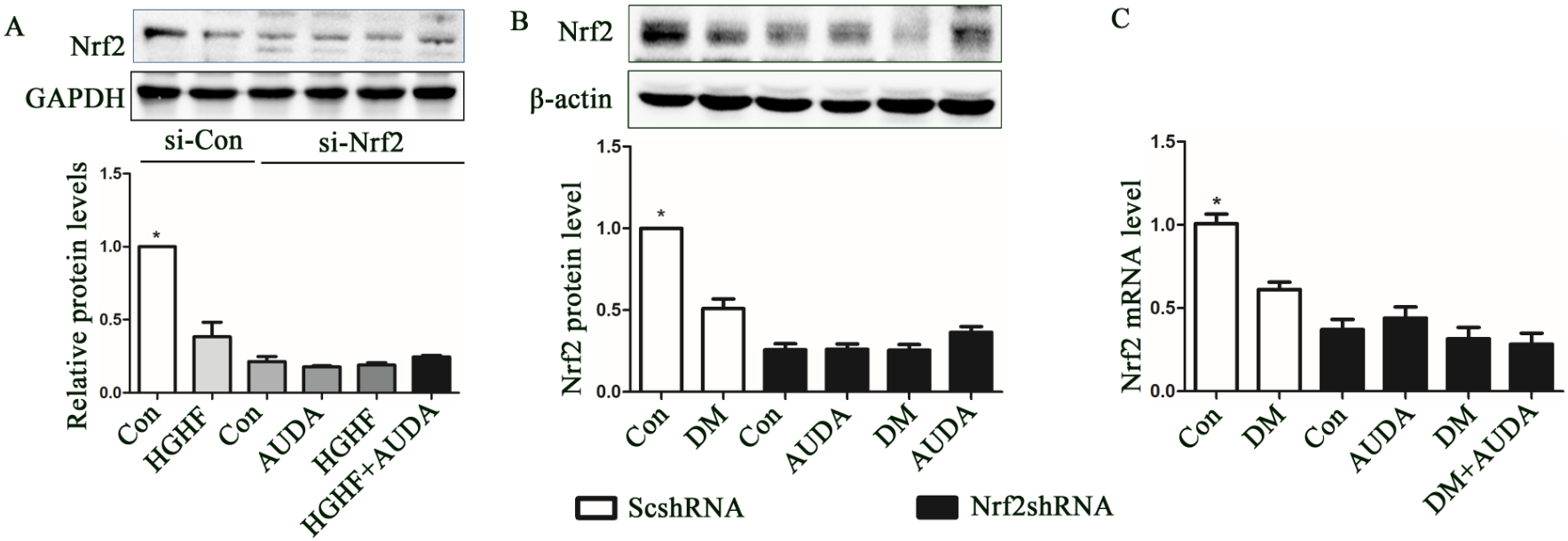


**Supplemental Figure 3. Small interfering RNA of Nrf2 and Virus carrying code for Nrf2 shRNA was worked in cardiomyocytes and mice heart.** (A) Representative immunoblots and quantitation of Nrf2 in cardiomyocytes in different groups; (B) Representative immunoblots and quantitation of Nrf2 in mice heart in different groups; (C) Nrf2 mRNA level was detected by qPT-PCR. Data were expressed as Mean ± SEM, n=8 mice per group, *p < 0.05 vs si-Con+Con or ScshRNA+Con. ScshRNA: AAV-ScshRNA; Nrf2shRNA: AAV-Nrf2shRNA; Con: db/m+vehicle or Control; AUDA: db/m+AUDA or AUDA; DM: db/db+vehicle.

Supplemental Figure 4


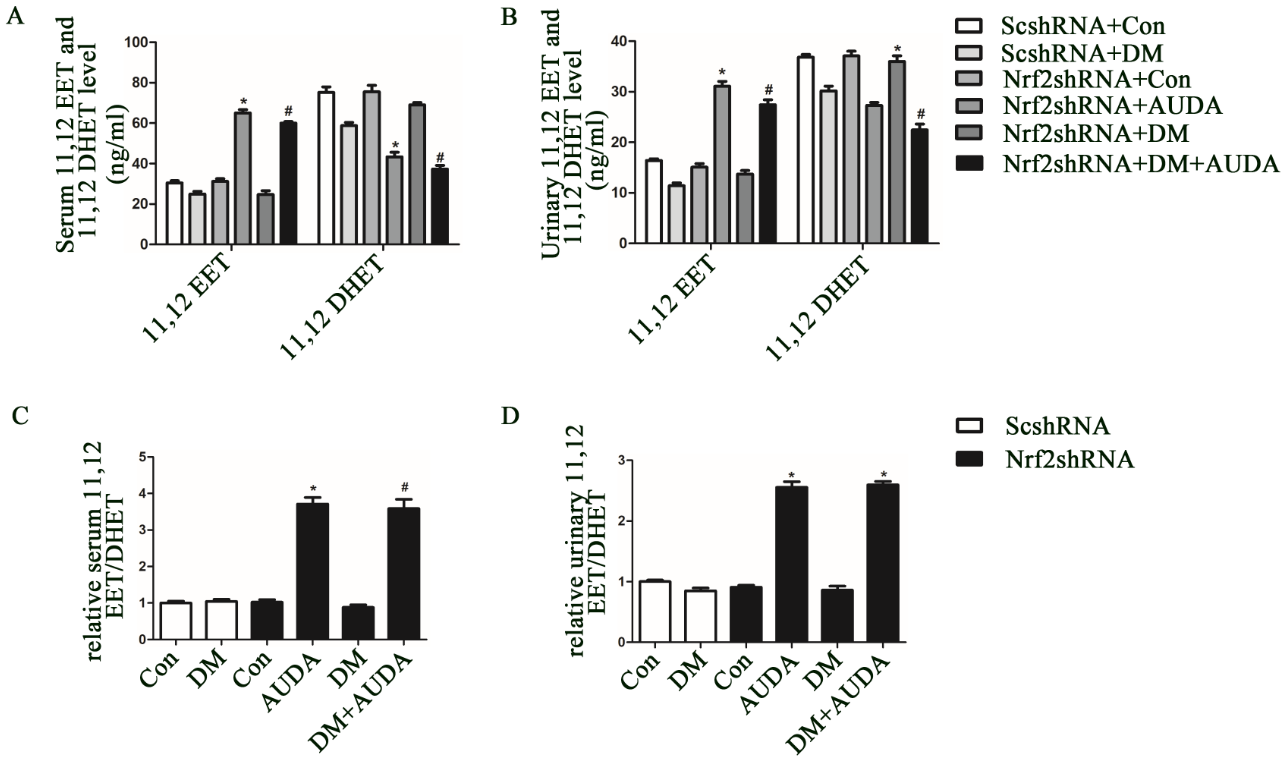


**Supplemental Figure 4. sEH inhibitor AUDA increased circulating EETs level in mice.** The concentration of 11, 12-EET and the corresponding 11, 12-DHET in serum (A) and urine (B) of mice which treated with AUDA. 11, 12 EET/DHET in serum (C) and urine (D) were calculated as index of sEH activity. Data were expressed as Mean ± SEM, n=8 mice per group, *p < 0.05 vs Nrf2shRNA+Con, #p < 0.05 vs Nrf2shRNA+DM. ScshRNA: AAV-ScshRNA; Nrf2shRNA: AAV-Nrf2shRNA; Con: db/m+vehicle; AUDA: db/m+AUDA; DM: db/db+vehicle.
